# Assessing the Dietary Practices and Anthropometric Outcomes of Students Consuming Mid-day Meals Versus Home-Cooked Meals

**DOI:** 10.7759/cureus.71110

**Published:** 2024-10-08

**Authors:** Adeeba Saleem, Sharmistha Goel, Mrinal Singh, Anmol Mathur, Anushka Choudhary

**Affiliations:** 1 Public Health Dentistry, Manav Rachna Dental College, Manav Rachna International Institute of Research and Studies, Faridabad, IND; 2 Dentistry, Manav Rachna Dental College, Manav Rachna International Institute of Research and Studies, Faridabad, IND

**Keywords:** dietary supplements, home-cooked meals, mid-day meal, nutritional status, social media impact

## Abstract

Introduction: Early childhood and adolescence are prime years that people spend in school. These formative years give the school ample opportunities to impart certain aspects of lifestyle and healthcare to the student, in addition to academics. School health programs are vital in providing comprehensive education and healthcare services. The objectives of the study are to enlist the socio-demographic factors affecting the nutritional level between students who enrolled for the mid-day meal scheme and those who did not.

Methodology: This is a cross-sectional questionnaire-based study, which was carried out between children who enrolled in the mid-day meal program and home-cooked meals for students from 3rd to 12th grade in Faridabad. The study was conducted by the Department of Public Health Dentistry, Faridabad, from January to June 2024.

Result: It was determined that 162 (53.6%) males and 222 (74.9%) females were obese. 332 (55.3%) believed that social media had an impact on their dietary decisions, while 268 (44.7%) disagreed. When asked to rate how many healthy food alternatives were available at their school, 53 (8.8%) of them said it was great, 194 (32.3%) said it was good, 196 (32.7%) said it was fair, and 157 (26.2%) said it was poor. 236 (78.7%) parents of the mid-day meal group never had dietary supplemental education, which is why children have indigent nutritional status. The mean waist-to-hip ratio (WHR) was calculated as 0.92 ± 0.43 and 0.88 ± 0.69 with significant values of 0.00 (p≤0.05) in the home-cooked and mid-day meal-consuming groups respectively.

Conclusion: Students consuming home-cooked meals showed a better WHR for age than those consuming mid-day meals. Children of parents with high education levels have good nutritional status and are healthier as compared to children with parents who have low education levels. Thus, there is a dire need to include school health programs to provide healthy and nutritious food to children, in need.

## Introduction

Early childhood and adolescence are the prime years that people spend in school. These formative years give the school ample opportunities to impart certain aspects of lifestyle and healthcare to the students, in addition to academics. Indeed, schools should be a priority in providing complete health education to their students during the young, tender years of school life, so they will get an all-around education on their health needs and live a healthier lifestyle [1]. School health programs are vital in providing comprehensive education and healthcare services. By partnering with various agencies, these programs seamlessly integrate multiple health components, thus creating a synergistic impact on overall health and well-being. Nutrition is among one of them. Eating habits and nutritional intake fundamentally determine growth and development. During school age, which encompasses childhood and adolescence, significant physical, and mental growth and development occurs [2].

A child needs to have proper nutritional intake to support healthy growth and development. By providing adequate nutrients, one can prevent the development of behavioral patterns that can either lead to obesity or nutritional deficiencies, which in turn can pose physiological risks and increase the likelihood of developing lifestyle disorders later in life. Research has demonstrated that school nutrition programs have a direct link with academic performance and play a crucial role in improving overall well-being. This improvement may be due to their positive impact on brain development, cognitive function, memory, attention, behavior, and attendance among students. A study by Frisvold mentioned the importance and role of micronutrients such as iron, zinc, vitamin B12, and more in long-term cognitive development [3]. They influence dopamine transmission and synthesis of neurotransmitters, which further affect memory and cognition.

Children with low levels of nutrition have been observed to suffer from both mental and physical developmental disabilities [4]. As a result, students suffer academically, attend school less frequently, are more likely to be tardy, and have a higher prevalence of illnesses that go undetected. This is especially true for students in lower-class neighborhoods who may have trouble affording food and finding a place to eat [5]. The nutritional analysis of students in private and government schools provides valuable insights into the disparities in dietary quality and its impact on health and academic performance.

Restricted growth as a result of inadequate nutrition may be measured using anthropometric status [6-9]. To determine dietary incompetency, measurements such as BMI, waist circumference (WC), and waist-to-hip ratio (WHR) are accessible and affordable [10]. By comparing the nutritional intake of students from these diverse educational settings, we can identify gaps and develop targeted interventions to promote equitable access to healthy foods [11].

In addition to shedding light on the sociodemographic differences between the two groups, the study attempts to evaluate the nutritional differences between students who enrolled for the mid-day meal scheme and those who do not. According to the study's hypothesis, there is no difference in the nutritional intake of the home-cooked and the mid-day meal groups. The aim of the study is to assess the dietary practices and anthropometric outcomes of students consuming mid-day meals versus home-cooked meals.

## Materials and methods

This is a cross-sectional, questionnaire-based study that was carried out amongst students from 3rd to 12th grades in Faridabad who enrolled in the mid-day meal scheme versus those who had home-cooked meals. The study was conducted by the Department of Public Health Dentistry, Faridabad, from January to June 2024. The purpose of the study was to evaluate the practices on nutrition consumption and measurement of their anthropometric measures.

The Department of Nutrition and Dietetics of Manav Rachna International Institute of Research and Studies collaborated to create a questionnaire with 21 English-language questions consisting of practices on nutritional intake. Please refer to Appendix 1 for the questionnaire. The face and content validity of the questionnaire was done by the Department of Nutrition and Dietetics and the final questionnaire was designed following the improvements. Initially, the questionnaire consisted of 27 questions, but after the final validation by 9 nutritionists from the Department of Nutrition and Dietetics, we only had 21 questions altogether because 7 questions were removed as they could not fulfill the minimum score of 0.78 of the Content Validity Ratio score (CVR) among 9 nutritionists. The pilot testing provided a prevalence of 54% nutritional deficiency among the school children, this when computed to the formula (n=4pq/d^2^) derived a sample of 276, this was rounded-off to 300 in each group. Ethical clearance was obtained from Manav Rachna Dental College Institutional Ethics Committee (Ref. no.: MRDC/IEC/2024/113).

Inclusion criteria

The mid-day meal group consisted of students in the age range of 8 to 18 who had signed up for the program and had been eating mid-day meals consistently for three years. The study participants were chosen from among the schools that responded to the invitation. In the study, parents/students who provided their written informed consent/assent were taken into account. Students who were present on the day of the commencement of study and had no medical history were included.

**Figure 1:**
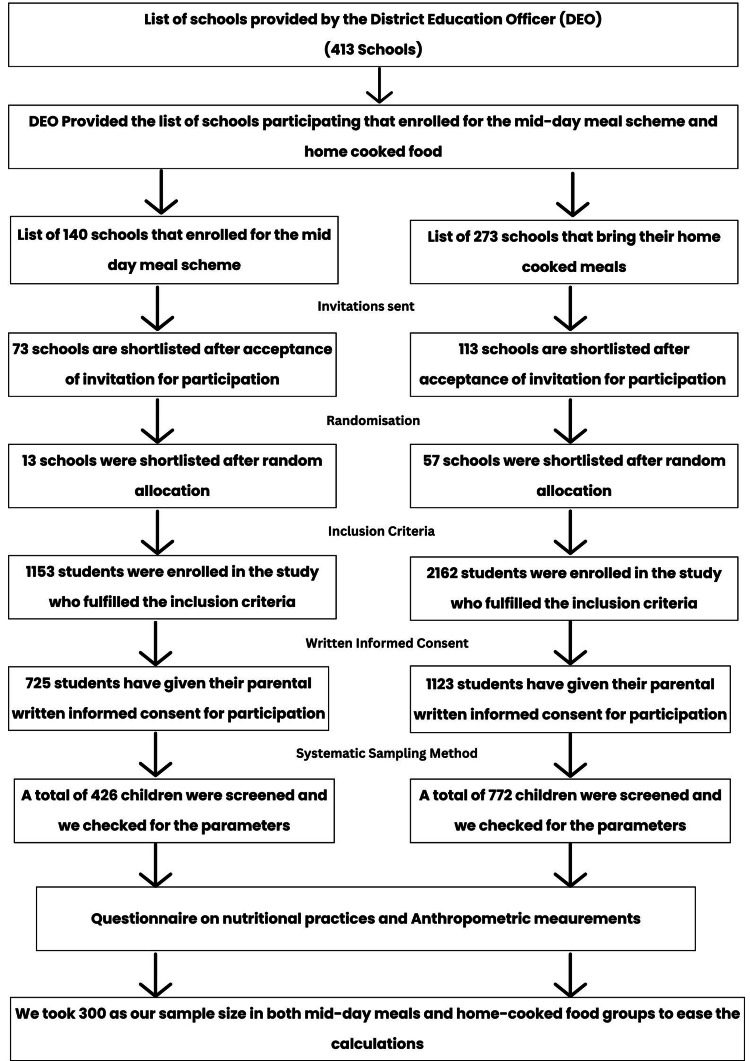
A step-by-step flowchart procedure for student selection

We selected the school from the list of schools provided by the District Education Officer (Letter no.: G-I/2022/1047) and the list of enrolled schools was selected after giving the necessary information about the commencement of the study. Before distributing the questionnaire, the written informed consent was taken from each participant. The objective of the study was described to the participants, and they were given the assurance that the information they submitted would be kept private and confidential. A questionnaire was filled out by interviewing method by the nutritionist. It consists of demographic information, such as age, sex, level of education of parents, and social class using Brahm Govind Prasad (B.G. Prasad) socioeconomic scale 2024 [12]. The study period was six months. The two groups were selected through flow charts mentioned in Figure 1.

All participating schools' students who were available during the visit were the data sources. The waist and hip values were measured according to WHO criteria, following their cut-off values [11]. Before starting at every new school, the measurement tool was checked for reliability and consistency. Prior to the visits, the schools were notified, and the parents' written informed consent was acquired via a note left on the children's homework diaries.

The data were exported and examined using Statistical Package for Social Sciences (SPSS) software version 27. The data was unevenly distributed among the population Mann-Whitney U test was run, and the p-value was maintained at 0.05.

## Results

This cross-sectional, questionnaire-based study was carried out among 3rd- to 12th-grade students in Faridabad, and 600 participants enrolled as a part of the mid-day meal and home-cooked meal group.

Table 1 shows 300 (50%) identified as male and 300 (50%) as female. In the research population, the parents who received primary education or were illiterate had similar level of education regarding dietary awareness; hence, their scores are clubbed. 67 (22.3%) fathers and 180 (60.3%) mothers from the mid-day meal scheme had completed their education till primary schooling. In comparison, 14 (4.7%) and 29 (9.7%) fathers had completed their post-graduation in mid-day meal and home-cooked groups, respectively, whereas 180 (60.3%) of the mothers were educated till primary class, 24 (8%) were post-graduate in the mid-day meal group. According to B.G. Prasad's updated scale 2024 for socioeconomic status on monthly per capita income, 171 (57%) of the home-cooked group belonged to the upper class, whereas 123 (41%) of the mid-day meal group belonged to the lower class.

**Table 1:** Distribution of sample according to age, sex and other demographic information

Demographic data	Options	Home-cooked food	Mid-day meal group	Frequency (percentage)
Age	8-13 years	205 (68.3)	217 (72.3)	422 (70.3)
	14-18 years	95 (31.7)	83 (27.7)	178 (29.7)
Sex	Male	159 (53)	139 (46.3)	300 (50)
	Female	139 (46.3)	161 (53.7)	300 (50)
Education of father	Illiterate-till 5th class	41 (13.6)	67 (22.3)	108 (18)
	Till 12th class	105 (35)	139 (46.3)	244 (40.7)
	Graduate	125 (41.7)	80 (26.7)	205 (34.2)
	Post-graduate	29 (9.7)	14 (4.7)	43 (7.2)
Education of mother	Illiterate-till 5th class	67 (22.3)	180 (60.3)	247 (41.2)
	Till 12th class	106 (35.3)	85 (28.3)	191 (31.8)
	Graduate	103 (34.3)	70 (23.3)	173 (28.8)
	Post-graduate	24 (8)	24 (8)	48 (16)
Social class	I	171 (57)	13 (4.3)	184 (30.6)
	II	111 (37)	27 (9)	138 (23)
	III	9 (3)	51 (17)	60 (10)
	IV	5 (1.6)	86 (28.6)	91 (30.3)
	V	4 (1.3)	123 (41)	127 (21.1)
Hip-waist ratio (male)	<0.96	55 (34.6)	81 (58.3)	136 (46.4)
	>0.96	104 (65.4)	58 (41.7)	162 (53.6)
Hip-waist ratio (female)	<0.83	17 (12)	61 (38.1)	78 (25.1)
	>0.83	122 (88)	100 (61.9)	222 (74.9)

It was determined that 162 (53.6%) males and 222 (74.9%) females were obese, respectively. 55 (34.6%) and 81 (58.3%) males had poor nutrition status in home-cooked and mid-day meal groups, respectively. 122 (88%) and 100 (61.9%) females were obese in home-cooked and mid-day groups, respectively.

Table 2 represents the study population. 249 (41.8%) had a mixed diet, and 349 (58.2%) followed a vegetarian diet. When asked how frequently they ate fast food or visited restaurants, 198 (33%) survey participants indicated that they did so more frequently, that is, 2-4 times per week, while just 62 (10.3%) reported doing so 2-4 times each month.

**Table 2:** Frequency and percentage of responses regarding nutritional practices

Questions	Options	Home-cooked group	Mid-day meal group	Frequency (percentage)
Type of diet	Vegetarian	167 (55.7)	182 (60.7)	349 (58.2)
	Mixed diet	133 (44.3)	118 (39.3)	249 (41.8)
How often do you eat fast food or go to a restaurant?	1 or less/week	87 (29)	75 (25)	162 (27)
	2-4/week	111 (37)	87 (29)	198 (33)
	0-1/month	67 (22.3)	111 (37)	178 (29.7)
	2-4/month	35 (11.7)	27(9)	62 (10.3)
How frequently do you exercise?	Daily/5 times a week	162 (55)	150 (50)	312 (52)
	2 days/week	84 (28)	88 (29.3)	172 (28.7)
	Never exercised	54 (18)	62 (20.7)	116 (19.3)
How would you rate the availability of healthy food options at your school?	Excellent	12 (4)	41 (13.7)	53 (8.8)
	Good	80 (26.7)	114 (38)	194 (32.3)
	Fair	101 (33.7)	95 (31.7)	196 (32.7)
	Poor	107 (35.7)	50 (16.7)	157 (26.2)
Do you take any dietary supplements?	Yes	88 (29.3)	72 (24)	160 (26.7)
	No	208 (69.3)	215 (71.7)	423 (70.5)
	Don’t know	4 (1.3)	13 (4.3)	17 (2.8)
Do you think social media influences your food choices?	Yes	192 (64)	140 (46.7)	332 (55.3)
	No	108 (36)	160 (53.3)	268 (44.7)
Do you snack between meals?	Yes	184 (61.3)	191 (63.7)	375 (62.5)
	No	116 (38.7)	109 (36.3)	225 (37.5)
Have your parents received any formal education about nutrition?	Yes	255 (85)	54 (18)	309 (51.5)
	No	23 (7.7)	236 (78.7)	259 (43.2)
	Maybe	22 (7.3)	10 (3.3)	32 (5.3)
Are your parents and guardians involved in planning and preparing meals at home?	Yes	162 (54)	193 (64.3)	355 (59.2)
	No	19 (6.3)	25 (8.3)	44 (7.3)
	Maybe	119 (39.7)	82 (27.3)	201 (33.5)

People who exercise five times a week and daily exhibit the same positive effects, therefore we combined the two scores to make the computation easier. When asked how frequently they exercised, 312 (52%) said they exercised every day or 5 days/week, while 288 (48%) said they did it less than 2 days/week. When asked to rate how many healthy food alternatives were available at their school, 53 (8.8%) of them said it was excellent, 194 (32.3%) said it was good, 196 (32.7%) said it was fair, and 157 (26.2%) said it was poor. Only 88 (29.3%) and 72 (24%) of them acknowledged taking dietary supplements in home-cooked and mid-day meal groups, respectively, and 228 (76%) and 212 (70.6%) of them denied using them in the mid-day meal and home-cooked groups, respectively. Of the participants in the study, 332 (55.3%) believed that social media impacted their dietary decisions, while 268 (44.7%) disagreed. Of the respondents, 375 (62.5%) snacked between meals, while only 225 (37.5%) didn't, among which 191 (63.7%) belong to the mid-day meal group. When asked if their parents or guardians help them plan and prepare meals at home, 355 (59.2%) answered they did, 201 (33.5%) said they might, and 44 (7.3%) said they didn't. 255 (85%) of the parents received formal education about nutrition in home-cooked food whereas 236 (78.7%) did not receive any education in the mid-day meal group.

After analyzing the average waist/hip ratio between mid-day meal and home-cooked food groups, we discovered that the mean for mid-day meal schools was 0.88 ± 0.69, and the average for home-cooked meal schools was 0.92 ± 0.43 (Table 3). When we applied the Mann-Whitney U test on schools offering mid-day meals and home-cooked food group meals, we found a significant value of 0.00 (p≤0.05), and when we compared the gender with the waist-hip ratio, it was found to be 0.04 (p≤0.05). In addition to mean, lower limit, upper limit, standard error difference, we also reported interquartile range (IQR) values.

**Table 3:** Mann-Whitney U test was applied for analysis of waist-hip ratio among mid-day meal and home-cooked food groups *: Significant, p-value ≤ 0.05 IQR: Interquartile range

		N	Lower limit	Upper limit	IQR	Mean and SD	Standard error difference	p-value
School type	Home-cooked food	300	0.13	1.72	0.38	0.92 ± 0.43	0.0047	0.00*
	Mid-day meal	300	0.18	1.9	0.52	0.88 ± 0.69		
Gender	Male	300	0.075	1.79	0.43	0.91 ± 0.06	0.005	0.04*
	Female	300	0.23	1.96	0.55	0.90 ± 0.06		

## Discussion

The study was conducted among the children who enrolled in the mid-day meal program and home-cooked meals from 3rd to 12th grades in Faridabad. The Department of Public Health Dentistry, Faridabad, conducted the study from January to June 2024. The study aimed to evaluate the anthropometric results and dietary habits of students who enrolled for the mid-day meals scheme compared to home-cooked meals.

According to WHO reports, the cut-off value for waist-hip ratio for obesity and overall risk is 0.83 for women and 0.96 for men [11]. According to our findings, 74.9% (222 participants) females of the total population were categorized as obese. A similar conclusion was reached in the research by Pelletier et al. and Ferretti et al. reported female counterparts as a twofold increased risk for being overweight, suggesting female dominance in overweight [9,13]. In addition, women have modality risk that is twice as high as compared to men and are more likely to develop medical and psychological comorbidities associated with obesity [13].

In our study, when we compared the mean waist-hip ratio between mid-day and home-cooked meals consuming schools, it had a highly significant value of 0.00, indicating that mid-day meal-providing schools had a lower calorific value than home-cooked food providing schools, and the supporting result was suggestive by Prakash et al. [14]. In contrast to our findings, the study by Souza et al. revealed that students consuming mid-day meal have more calories in the afternoon than their home-cooked food consuming counterparts, leading to higher levels of attentiveness in the classroom [15]. When comparing the average calorie intake of the government school's mid-day meals (357.6) and the home-cooked food of private-school children (306.6), the mean caloric intake for mid-day meals was greater. Another aspect of the enhanced nutritional value of a mid-day meal is that it is freshly prepared, meaning it is less likely to be heavy in calories and is lighter in oil than home-cooked food, which is more likely to contain high-calorie preparations that are meant to enhance the flavor even when they are served cold (e.g., sandwiches, noodles, puris, etc.).

Compared to their peers in the higher socioeconomic class, children from lower socioeconomic classes had lower nutritional status. The reason for poor nutritional status was that the mid-day meal program enrolled students from government schools who belonged to a low socioeconomic class. In contrast, the home-cooked meal group studying in private schools belonged to a higher socioeconomic class. A similar result was found by Babar et al. [4].

In total, 600 schools participated in our study: 300 (50%) offered mid-day meals, and 300 (50%) served home-cooked meals, among which 55 (34.6%) and 81 (58.3%) were underweight in home-cooked and mid-day meals, respectively. Similarly, according to Kapoor et al., there was a higher incidence of overweight, i.e., 66.85% in home-cooked food consumers, and in mid-day meal consuming schools, underweight students accounted for 14% of cases [16].

Dietary supplements are necessary for healthy growth and development and for achieving proper milestones [17,18]. In our study, 160 (26.7%) of the population took dietary supplements to meet their nutrient needs, whereas 440 (73.3%) did not take any supplements to ensure they got the recommended amount of nutrients. Our study showed that 236 (78.7%) of parents of the mid-day meal group never had dietary supplemental education, and this is why children have indigent nutritional status: 27 (9%) fathers and 63 (21%) mothers were illiterate, similar to the study by Das et al. that stated that children whose parents had only completed secondary school or less had a reduced nutritional supplements use (p<0.001) [19]. Of children whose mothers were working in the medical field, 44% utilized supplementary products, whereas 41.3% did not use any.

Children from wealthy and typical families supplemented their diets significantly more than those from low-income homes (73.7%, 33.6%, and 16.8%, respectively) because 255 (85%) parents received formal education about nutrition in home-cooked food and could afford the supplementary diet due to their high socioeconomic status. A similar result was found by Das et al. [19].

This study reported that 332 (55.3%) believed that social media influences their eating habits, while 268 (44.7%) disagreed. Similar findings were found in some studies that linked social media exposure to higher frequency of consumption of unhealthy snacks, fast food, and sugar-filled drinks; daily intake of caffeine and sugar; preference for fast food; and increased likelihood of skipping breakfast. The influence of social media on children's eating habits is a growing concern, especially with the rise of influencer marketing. Studies show that when popular social media influencers promote unhealthy foods, it significantly increases children's immediate food intake [20,21]. A contradictory result was found by Sina et al. [22]. Additionally, educating children and families about the impact of marketing could help build more awareness and critical thinking around food choices.

Limitations

The study was a cross-sectional study that only included participants from one region, so it may not represent all children in India of the same age group. Our study included a limited sample size. To enhance the study results among the study population, including testing methods such as hemoglobin testing and arm circumference would have been beneficial.

## Conclusions

After examining the data, it was found that students consuming home-cooked meals showed a better WHR for age compared to those consuming mid-day meals. Children of parents with high education levels have good nutritional status and are healthier as compared to children with parents who have low education levels. These insights are valuable for policymakers and education stakeholders in improving nutritional and health measures among adolescents.

## Appendices

Appendix 1

Questionnaire

A: Demographic data:

1.     Name:

2.     Age:

3.     Sex:

a.     Male

b.     Female

c.     Other

4.     Class/standard:

5.     Education of father:

a.     Illiterate

b.     5th class

c.     12th class

d.     Graduate

e.     Post-graduate

6.     Education of mother:

a.      Illiterate

b.     5th class

c.      12th class

d.     Graduate

e.      Post-graduate

7. School Type:

i.       Government: Mid-day meal group Home-cooked group

ii.      Private: Mid-day meal group Home-cooked group

8.     Hip (inches):

9.     Waist (inches):

10.  Social class:

a.      I

b.      II

c.      III

d.      IV

e.      V

B: Questionnaire on practice of nutrition intake

11.  Type of diet

a.      Vegetarian

b.      Mixed

12.  How often do you eat fast food or go to a restaurant?

a)     1 or less/week

b)     2-4/week

c)     0-1/month

d)     2-4/month

13.  How frequently do you exercise?

a.      Daily

b.     5 days/week

c.     2 days/week

d.     Not exercising

14.  Do you take any dietary supplements?

a.     Yes

b.     No

c.     Don't know

15.  Do you think the social media influences your food choices?

a.     Yes

b.     No

16.  Do you snack between meals?

a.     Yes

b.     No

17.  Have your parents received any formal education about nutrition?

a.     Yes

b.     No

c.     Maybe

18.  Are your parents and guardians involved in planning and preparing meals at home?

a.      Yes

b.      No

c.      Maybe

19.  How frequently do you feel hungry?

a.     Never

b.     Rarely

c.     Sometimes

d.     Often

e.     Always

20. Which of these meals or snacks did you eat yesterday? (Check all that apply)  

a.    Breakfast

b.    Lunch

c.    Dinner/supper

d.    Morning snack

e.    Afternoon snack

f.     Evening/late snack

21. How would you rate the cleanliness and ambiance of your school's dining area?

a.    Excellent

b.    Very good

c.    Good

d.    Fair

e.    Poor
